# Steam and Flame Applications as Novel Methods of Population Control for Invasive Asian Clam (*Corbicula fluminea*) and Zebra Mussel (*Dreissena polymorpha*)

**DOI:** 10.1007/s00267-020-01325-1

**Published:** 2020-07-05

**Authors:** Neil E. Coughlan, Eoghan M. Cunningham, Stephen Potts, Diarmuid McSweeney, Emma Healey, Jaimie T. A. Dick, Gina Y. W. Vong, Kate Crane, Joe M. Caffrey, Frances E. Lucy, Eithne Davis, Ross N. Cuthbert

**Affiliations:** 1 Institute for Global Food Security, School of Biological Sciences, Queen’s University Belfast, 19 Chlorine Gardens, Belfast, BT9 5DL Northern Ireland UK; 2 Queen’s Marine Laboratory, Queen’s University Belfast, 12-13 The Strand, Portaferry, BT22 1PF Northern Ireland UK; 3 School of Biological, Earth and Environmental Sciences, University College Cork, Distillery Fields, North Mall, Cork, Ireland; 4 INVAS Biosecurity Ltd., 82 Lakelands Close, Stillorgan, Co Dublin, Ireland; 5 Centre for Environmental Research, Innovation & Sustainability, Institute of Technology Sligo, Ash Lane, Co, Sligo, Ireland; 6 GEOMAR, Helmholtz-Zentrum für Ozeanforschung Kiel, Kiel, Germany

**Keywords:** Invasive alien species, Biosecurity, Open-flame heat torch, Thermal shock, Population control, Eradication

## Abstract

Control strategies for established populations of invasive alien species can be costly and complex endeavours, which are frequently unsuccessful. Therefore, rapid-reaction techniques that are capable of maximising efficacy whilst minimising environmental damage are urgently required. The Asian clam (*Corbicula fluminea* Müller, 1774), and the zebra mussel (*Dreissena polymorpha* Pallas, 1771), are invaders capable of adversely affecting the functioning and biodiversity of freshwater ecosystems. Despite efforts to implement substantial population-control measures, both species continue to spread and persist within freshwater environments. As bivalve beds often become exposed during low-water conditions, this study examined the efficacy of steam-spray (≥100 °C, 350 kPa) and open-flame burn treatments (~1000 °C) to kill exposed individuals. Direct steam exposure lasting for 5 min caused 100% mortality of *C. fluminea* buried at a depth of 3 cm. Further, combined rake and thermal shock treatments, whereby the substrate is disturbed between each application of either a steam or open flame, caused 100% mortality of *C. fluminea* specimens residing within a 4-cm deep substrate patch, following three consecutive treatment applications. However, deeper 8-cm patches and water-saturated substrate reduced maximum bivalve species mortality rates to 77% and 70%, respectively. Finally, 100% of *D. polymorpha* specimens were killed following exposure to steam and open-flame treatments lasting for 30 s and 5 s, respectively. Overall, our results confirm the efficacy of thermal shock treatments as a potential tool for substantial control of low-water-exposed bivalves. Although promising, our results require validation through upscaling to field application, with consideration of other substrate types, increased substrate depth, greater bivalve densities, non-target and long-term treatment effects.

## Introduction

Invasive alien species can negatively impact ecological and evolutionary dynamics, often with adverse ecosystem and socioeconomic impacts (Simberloff et al. 2013; Sousa et al. 2014; Seebens et al. 2017). Currently, management options for eradication and control of established invader populations are often resource-intensive and costly processes (Caffrey et al. 2011b; Wittmann et al. 2012a, b; Piria et al. 2017; Coughlan et al. 2018b). The implementation of population-control strategies can be multifaceted and complex endeavours that require various challenges to be overcome. For instance, obstacles preventing the success of control strategies can include poor educational outreach and public engagement (Davis et al. 2018), legal impediments to accessing private property, regulatory issues concerning the provision of adequate resources to enforce legislation (Caffrey et al. 2014), a lack of cost-effective and repeatable management strategies (Aschim and Brook 2019) and limited ongoing monitoring (Piria et al. 2017). Further, the effectiveness of many potential eradication and population-control techniques is inadequate or unknown, and can be damaging to non-target species (Wittmann et al. 2012a, b; Luoma et al. 2018; Coughlan et al. 2019b). Therefore, innovative and accessible methods, which maximise efficacy of treatment towards target species whilst minimising broad-scale environmental damage, are urgently required (Coughlan et al. 2018a, b; Cuthbert et al. 2018, 2019).

Many invasive alien bivalves, as dominant filter feeders, can represent a major threat to the function and biodiversity of freshwater ecosystems (Higgins and Vander Zanden 2010; Sousa et al. 2014; Karatayev et al. 2015). For example, invasive bivalves can dominate macroinvertebrate communities and physically alter benthic habitats (Sousa et al. 2008; Karatayev et al. 2015). In particular, the Asian clam (*Corbicula fluminea* Müller, 1774) (Bivalvia, Cyrenidae, formerly Corbiculidae), and the Zebra mussel (*Dreissena polymorpha* Pallas, 1771) are both considered to be high-impact invaders that can dominate macroinvertebrate communities, physically alter benthic habitats and modify community and ecosystem dynamics through their propensity to form dense benthic beds (Sousa et al. 2008, 2014; Karatayev et al. 2015). Further, these freshwater invaders often display a high degree of physiological and ecological plasticity (Sousa et al. 2014), and have a remarkable capacity for human-mediated and even zoochorous dispersal (Belz et al. 2012; Coughlan et al. 2017b). In addition, infestations of both species can have substantial negative economic impacts, through macrofouling of agricultural, municipal and water extraction systems, increased sedimentation rates and the disruption of ecosystem-regulating services, sometimes resulting in the closure of sport fisheries and amenity areas (Karatayev et al. 2007; Nakano and Strayer 2014; Sousa et al. 2014). Nevertheless, these bivalves are occasionally associated with positive effects, primarily as an additional food source, as biomonitors or for the biofiltration of pollutants (e.g., Sousa et al. 2014), and these positive effects should be considered when designing management protocols.

Despite repeated management efforts to reduce their spread, both these species continue to advance across hydrologically unconnected freshwater systems worldwide (e.g., Caffrey et al. 2011a, 2016; Barbour et al. 2013). Once established, such bivalves can form dense populations (Sheehan et al. 2014; Hetherington et al. 2019) that cover extensive areas (e.g., up to 12,000 m^2^), which may become exposed during low tides and low river-flow conditions (Caffrey et al. 2016). Moreover, the current rate of climate change is predicted to increase the availability of suitable habitats for invasive bivalves within new river basins, especially at higher latitudes (Gama et al. 2017). Accordingly, a mosaic of freshwater environments are susceptible to the introduction and establishment of these invasive bivalves, which can subsequently act as new source locations that facilitate further spread (Sousa et al. 2014; Karatayev et al. 2015). Therefore, there remains an urgent need to develop and validate a suite of tools for rapid population control and eradication of emerging and existing populations (Colwell et al. 2017; Coughlan et al. 2018b, 2019b). To date, although extensive population-control experiments have been conducted on both *C. fluminea* (Wittmann et al. 2012a, b; Sheehan et al. 2014) and *D. polymorpha* (Luoma et al. 2018), none of them have been successful in providing substantial long-term management of these invaders.

Recently, with a series of laboratory experiments, Coughlan et al. (2018b) observed that cold thermal shock treatments, via the application of dry-ice pellets (i.e., solid CO_2_ pellets at −78 °C), could be used to kill tidally exposed and water-submerged *C. fluminea*. Similarly, Coughlan et al. (2019b) demonstrated that an open-flame torch (~1000 °C, i.e., hot thermal treatments) can also be used to kill mud-dwelling *C. fluminea*. Overall, these studies highlight the potential use of these novel methods for effective, rapid-response control and possible localised eradication of *C. fluminea* populations. Further, as part of biosecurity-decontamination protocols, several recent studies have highlighted the use of steam spray as an effective, yet straightforward user- and environmentally friendly mechanism for causing mortality of invasive alien species, including bivalves (Crane et al. 2019; Joyce et al. 2019; Coughlan et al. 2020). In particular, Coughlan et al. (2019a) documented that 30 s of direct steam exposure can consistently kill small groups of adult Asian clams. Accordingly, steam-induced thermal shock could represent an effective and environmentally friendly means of achieving substantial, if not complete, mortality of *C. fluminea* populations residing upon and within exposed lake, river or canal beds. In addition, innovative thermal shock treatments, such as steam and open flame, could potentially be used to facilitate substantial long-term population control of problematic *Dreissena* mussel species.

In the present study, using simulated bivalve beds, we examined the efficacy of thermal shock treatments to kill adult *C. fluminea* and *D. polymorpha*. We hypothesised that exposure to the extreme heat of steam spray or open flame will result in substantial, if not complete, mortality of both bivalve species due to thermal shock. In doing do, we assessed several key experimental factors: (1) direct exposure to steam, (2) indirect exposure to steam for buried specimens, (3) exposure to steam and open-flame burns following mechanical disruption of surrounding substrate, (4) longer thermal shock exposure times, (5) increased substrate depth and (6) water-submerged beds. Only direct applications were considered for *D. polymorpha*, which will only reside on top of substrate.

## Methods

### Specimen Collection and Maintenance

In May 2018, specimens of *C. fluminea* (>5000) were collected by hand from the extensive, tidally exposed area at Poulmounty on the River Barrow in the Republic of Ireland (52° 29′ 15.11″ N, 6° 55′ 42.20″ W), and transported in source water to Queen’s University Marine Laboratory (QML), Portaferry, Northern Ireland. Similarly, in May 2018, *D. polymorpha* specimens (>600) were collected by hand from Lough Erne, Northern Ireland (54° 17′ 07.89″ N, 7° 32′ 52.61″ W), and transported in source water to QML. In the laboratory, specimens were housed within a controlled temperature (CT) room at 13 °C, on a 12:12-h light-to-dark schedule. All specimens were maintained in aerated aquaria using locally sourced lake water (Lough Cowey: 54° 24′ 41.79″ N, 5° 32′ 25.96″ W). Specimens were allowed to acclimatise to the laboratory for at least 1 week prior to experimentation.

Only living and feeding specimens (i.e., selected specimens that were observed opening to feed, and which closed when disturbed) were selected for experimental work. Adult *C. fluminea* and *D. polymorpha* specimens were selected based on shell height (SH) or shell length (SL), for each species, respectively. Following completion of this study, ≥1000 *C. fluminea* and ≥200 *D. polymorpha* specimens not used for experiments were separately maintained within the aquaria for over a 3-month period. For both species, greater than 95% survival of these specimens was observed.

Following experimental exposure, all specimens were allowed to air-cool for 15 min, including control groups. All groups were then returned to the CT room, with replicates placed individually in 600 ml of dechlorinated tap water taken from a continuously aerated source (11–13 °C) for a 24-h recovery period, after which mortality was assessed. Specimens were considered dead if they were gaping, or failed to respond to a tactile stimulus, such as being teased apart with tweezers (see Matthews and McMahon 1999).

### Experiment 1: Direct Steam Exposure for Inducing *Corbicula fluminea* Mortality

*C. fluminea* specimens (SH min.–max.: 18–20 mm) were directly exposed to a continuous jet of steam (≥100 °C, 350 kPa, Karcher^®^ SC3 Steam Cleaner) at a distance of 2–3 cm from the exit point of the lance for 10, 30, 60 or 120 s. All treatment combinations were replicated in triplicate, i.e., *n* = 3. Groups of ten adult specimens were selected from the aquaria and randomly positioned onto a 6-cm-deep bed of dry sand, within cylindrical plastic containers of 234 mm (height) × 180 mm (diameter), producing a density of 393 ind. m^–2^. Specimens were then gently pressed into the sand until half of each specimen was exposed. Control groups were, likewise, pressed into the sand and allowed to air-dry for the longest exposure time of 120 s, and these specimens were not exposed to steam.

### Experiment 2: The Effectiveness of Direct and Indirect Steam Exposure for Inducing *Corbicula fluminea* Mortality

*C. fluminea* specimens (SH: 18–20 mm) were exposed directly or indirectly to steam spray for 1, 2, 3 or 5 min. All treatment combinations were replicated in triplicate, i.e., *n* = 3. Groups of 30 *C. fluminea* (1179 ind. m^−2^) were placed randomly within the cylindrical experimental containers upon a 6-cm-deep bed of dry sand. Specimens were gently pressed into the sand until half of each individual was exposed. For indirect exposure groups, an additional 3 cm of dry sand was used to cover the specimens entirely. Control groups were likewise placed into containers and covered with sand, as required by the experimental design. All control groups were allowed to air-dry for the longest exposure time of 5 min, and these specimens were not exposed to steam.

### Experiment 3: Efficacy of Combined Rake and Steam Treatments for Inducing *Corbicula fluminea* Mortality

As burial of *C. fluminea* within substrate appears to limit the efficacy of steam-spray treatments (see “Results”), the combined application of rake and steam was examined. The initial raking phase was used to churn up the substrate in order to expose greater numbers of *C. fluminea* to the subsequent application of steam. Specimens of *C. fluminea* (SH: 20–22 mm) were exposed to steam-spray treatments for 2.5 min, following a 30-s period of patch raking (Fiskars soil rake). The combined application of rake and steam was examined for single, double and triple treatments. All treatment combinations were replicated in triplicate, i.e., *n* = 3. Groups of 30 *C. fluminea* (480 ind. m^–2^) were randomly mixed into a damp-sand layer to create a simulated patch of sand-dwelling bivalves, which is representative of a realistic *C. fluminea* bed structure (25 cm × 25 cm, *~* 4-cm deep). Control groups were, likewise, formed into sand patches, which were each raked for three consecutive 30-s periods and allowed to air-dry for 2.5 min following each raking event. Control patches were not exposed to steam.

### Experiment 4: Efficacy of Rake and Steam Treatments or Rake and Burn Treatments for Inducing *Corbicula fluminea* Mortality Following Longer Exposure Periods

Given that complete mortality of sand-dwelling *C. fluminea* was not observed, even following three consecutive rake and steam applications (see “Results”), longer steam and open-flame burn application periods were examined. Specimens of *C. fluminea* (SH: 21–23 mm) were exposed to steam-spray (≥100 °C, 350 kPa) or open-flame treatments (~1000 °C, 400 kPa: Rothenberger, Romaxi Power burner) for a 5-min period, following a 30-s period of patch raking. This combined application of rake and thermal treatment was examined for single, double and triple treatments. All treatment combinations were replicated in triplicate, i.e., *n* = 3. Full personal protection gear was used by all operatives and observers, with flame-retardant foam on standby following a visual risk assessment (S. Exley *pers. comm*.). As before, 30 *C. fluminea* (480 ind. m^–2^) were randomly mixed through damp sand to create simulated patches (25 cm × 25 cm, ~4-cm deep). Control groups were likewise formed into sand patches, and these control patches were raked for three consecutive 30-s periods and allowed to air-dry for a 5-min period following each raking event. Control patches were not exposed to thermal treatments.

### Experiment 5: Efficacy of Rake and Steam or Rake and Burn Treatments for Inducing *Corbicula fluminea* Mortality at Increased Substrate Depth

To further examine the potential buffering effect of substrate, an additional depth of ~8 cm of sand was used. Following a 30-s period of patch raking, specimens of *C. fluminea* (SH: 22–24 mm) were exposed to steam-spray or open-flame treatments for a 5-min period. This combined application of rake and thermal treatment was examined for single, double and triple treatments. All treatment combinations were replicated in triplicate, i.e., *n* = 3. As before, 30 *C. fluminea* (480 ind. m^−2^) were randomly mixed through damp sand to create simulated patches (25 cm × 25 cm). As before, control groups were likewise formed into patches, which were consecutively raked and air-dried for a 5-min period, three times overall. These patches were not exposed to thermal treatments.

### Experiment 6: Efficacy of Rake and Steam Treatments for Inducing Mortality of Water-Submerged *Corbicula fluminea*

To assess the effect of submersion in water, sand-dwelling *C. fluminea* was exposed to steam through a 1-cm layer of water. Specimens of *C. fluminea* (SH: 23–25 mm) were exposed to steam- spray treatments for a 5-min period, following a 30-s period of patch raking. This application of combined rake and steam treatments was examined for single, double and triple treatments. All treatment combinations were replicated in triplicate, i.e., *n* = 3. Groups of 30 *C. fluminea* (480 ind. m^−2^) were randomly mixed through damp sand to create simulated patches (25 cm × 25 cm, ~4-cm deep). Simulated patches were constructed within plastic trays (80*L* × 50*W* × 6*H* cm). Tap water was then added until a 1-cm layer was created between the patch and the water surface. Control groups were likewise formed into submerged patches, which were raked, then left undisturbed for a 5-min period and repeated three times overall. Control patches were not exposed to steam.

### Experiment 7: Direct Steam Exposure for Inducing *Dreissena polymorpha* Mortality

*D. polymorpha* specimens were exposed to steam-spray applications for 5, 10, 30, 60 or 120 s. All treatment combinations were replicated in triplicate, i.e., *n* = 3. Groups consisting of 30 *D. polymorpha* (SL mean ± SE, min.–max.: 22.13 ± 0.1, 16.9–30.08 mm) were briefly maintained in dechlorinated tap water (<30 min) and extracted as needed. Groups were placed as loose clump on flat-gravel patches (15-mm stone chips) and exposed to steam spray. Control specimens were allowed to air-dry for a 1-min period, and were not exposed to steam spray.

### Experiment 8: Direct Open-flame Exposure for Inducing *Dreissena polymorpha* Mortality

*D. polymorpha* specimens were directly exposed to open-flame applications for either 5 or 10 s. All treatment combinations were replicated in triplicate, i.e., *n* = 3. Groups of 30 *D. polymorpha* (SL: 22.57 ± 0.2, 16.9–28.68 mm) were maintained in dechlorinated tap water (<30 min) and extracted as needed. Groups were placed as loose clump on flat-gravel patches (15-mm stone chips) and exposed to open flame for 5 or 10 s. Control specimens were allowed to air-dry for a 1-min period, and were not exposed to open flame.

### Data Analyses

Binomial generalised linear models with logit links were used to examine bivalve mortality rates with respect to experimental treatment for each experiment separately. For examination of direct and indirect steam-exposure efficacies (i.e., Experiment 2), cover (2 levels) was additionally included as a single and interacting term to denote direct and indirect treatment efficacies. The mean bias-reducing adjusted score approach was implemented owing to complete separation in cases of total bivalve mortality and/or survival (Firth 1993; Kosmidis 2014). Analysis of deviance was used for inferences of the main effect sizes, with Type II sums of squares implemented for single terms, and Type III sums of squares in the presence of a significant interaction (Fox and Weisberg 2011). Estimated marginal means with Tukey adjustments were used for post hoc treatment-level contrasts where an effect was significant (Lenth 2018). All statistical analyses were performed in R v3.5.1 (R Core Development Team 2018).

## Results

### Experiment 1: Direct Steam Exposure for Inducing *Corbicula fluminea* Mortality

**Fig. 1:**
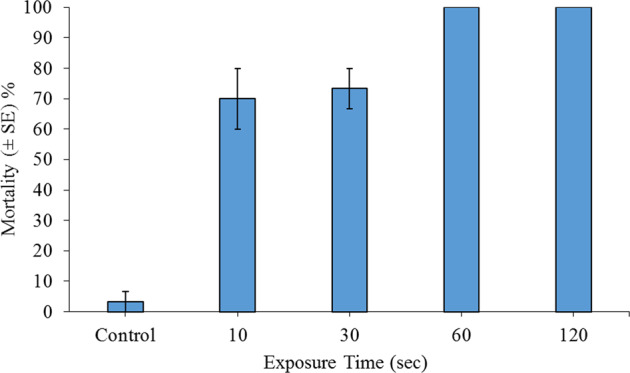
Mean mortality (±SE) of ten adult *Corbicula fluminea* specimens (393 ind. m^–2^) 24 h post exposure to direct steam spray, for up to 120 s (≥100 °C). All treatment combinations were replicated in triplicate, i.e., *n* = 3. All clams were partially buried while residing upon the surface of dry-sand substrate, i.e., half of each specimen was buried by sand

There was between 90 and 100% (i.e., min.–max.) survival of control specimens, while 100% mortality of *C. fluminea* was consistently observed following steam exposures of 60 and 120 s (Fig. 1). Steam exposure significantly influenced bivalve mortality (GLM: *χ*^2^ = 102.58, df = 4, *P* < 0.001), with steam always driving significantly greater mortality than controls (all *P* < 0.001). Differences among steam-exposure durations were not statistically clear (all *P* > 0.05).

### Experiment 2: The Effectiveness of Direct and Indirect Steam Exposure for Inducing *Corbicula fluminea* Mortality

**Fig. 2:**
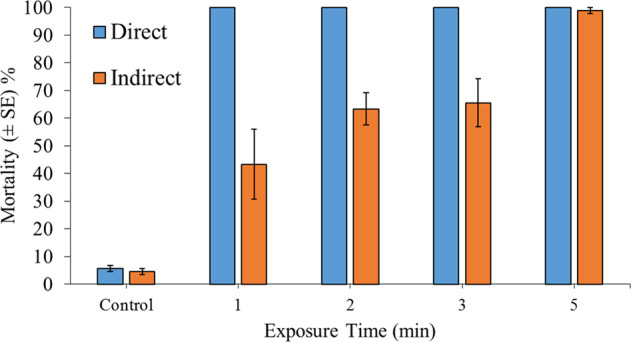
Mean mortality (±SE) of 30 adult *Corbicula fluminea* specimens 24 h post direct (partially buried, i.e., half of each specimen was buried by dry-sand substrate) or indirect (fully buried below 3 cm of dry sand) exposure to steam-spray treatments, for up to 5 min, respectively. All treatment combinations were replicated in triplicate, i.e., *n* = 3. In both cases, *C. fluminea* density is 1179 ind. m^−2^

Between 93 and 97% survival was documented for control specimens, for both directly and indirectly treated groups. Direct steam exposures lasting for 1 min or longer always caused 100% mortality of *C. fluminea*, whilst mortality rates between 30.7 and 100% were observed following indirect exposures, with increased exposure times resulting in greater mortality (Fig. 2). The steam- treatment effect thus interacted significantly with the level of cover (GLM: *χ*^2^ = 22.95, df = 4, *P* < 0.001), reflecting greater differences among steam exposures following indirect treatments. In particular, indirect 5-min exposures were more efficacious than all other durations (all *P* < 0.001), whilst 3-min indirect exposures drove significant mortality compared with 1-min durations (*P* = 0.03). Differences between 1- and 2-min indirect exposures and 2- and 3-min indirect exposures were not statistically clear (both *P* > 0.05). Nevertheless, both direct and indirect steam treatments always induced significant mortality, compared with controls (all *P* < 0.001).

### Experiment 3: Efficacy of Combined Rake and Steam Treatments for Inducing *Corbicula fluminea* Mortality

**Fig. 3:**
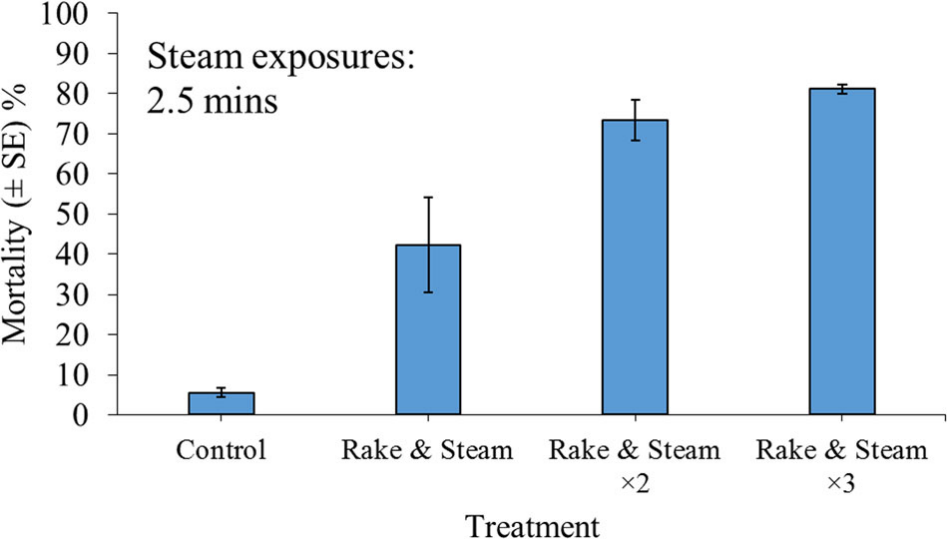
Mean mortality (±SE) of 30 adult *Corbicula fluminea* specimens (480 ind. m^–2^) 24 h post exposure to the application of combined 30-s rake and 2.5-min steam-spray treatments while residing within a 4-cm-deep patch of damp-sand substrate, performed as a single, double (×2) or triple (×3) treatment. All treatment combinations were replicated in triplicate, i.e., *n* = 3

There was between 93 and 97% survival of control specimens, while up to 83% mortality was observed following triple applications of rake and steam treatment (Fig. 3). Single and double applications caused lower mortality. The rake and steam treatment was significant in inducing *C. fluminea* mortality (GLM: *χ*^2^ = 146.16, df = 3, *P* < 0.001). Double and triple applications were more efficacious than single rake and steam applications (both *P* < 0.001), whilst differences between double and triple treatments were less clear statistically (*P* = 0.61). However, significant mortality was observed following all treatments compared with controls (all *P* < 0.001).

### Experiment 4: Efficacy of Rake and Steam Treatments or Rake and Burn Treatments for Inducing *Corbicula fluminea* Mortality Following Longer Exposure Periods

**Fig. 4:**
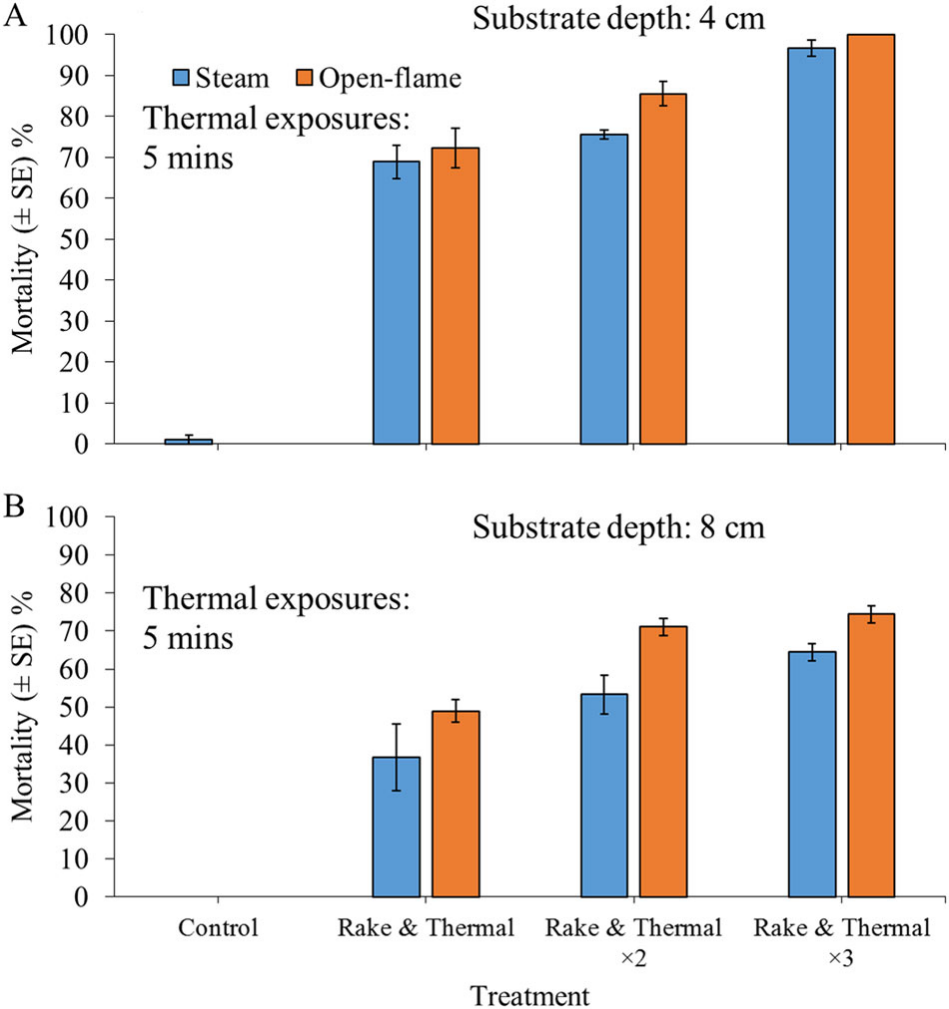
Mean mortality (±SE) of 30 adult *Corbicula fluminea* specimens (480 ind. m^–2^) 24 h post exposure to the application of combined 30-s rake and 2.5-min thermal shock treatments, while residing within damp- sand substrate at a depth of **a** 4 cm and **b** 8 cm. Thermal treatments consisted of steam-spray or open-flame exposure, i.e., ≥100 °C or ~1000 °C, respectively. All treatments were performed as single, double (×2) or triple (×3) applications. All treatment combinations were replicated in triplicate, i.e., *n* = 3

Between 97 and 100% survival of control specimens was recorded. An average of 97% *C. fluminea* mortality was exhibited after triple rake and steam treatments, whilst 100% mortality was always observed following triple rake and burn applications (Fig. 4a). For both treatment types, single and double applications caused lower mortality. Treatments caused significant differences in mortality for *C. fluminea* (GLM: *χ*^2^ = 322.88, df = 6, *P* < 0.001). There were no significant differences in mortality between steam and burn applications under matched single, double or triple applications (all *P* > 0.05). However, open-flame burn treatments tended to kill more *C. fluminea* than steam applications. All treatments caused significantly higher mortality, compared with control groups (all *P* < 0.001).

### Experiment 5: Efficacy of Rake and Steam or Rake and Burn Treatments for inducing *Corbicula fluminea* Mortality at Increased Substrate Depth

There was 100% survival of control specimens, while up to 67% and 77% *C. fluminea* mortality was observed following both triple applications of rake and steam, and triple applications of rake and burn treatments, respectively (Fig. 4b). Significant differences in mortality were recorded among treatment groups (GLM: *χ*^2^ = 177.32, df = 6, *P* < 0.001). As with Experiment 4, differences between steam and burn treatments did not differ significantly at matched application types (all *P* > 0.05). However, once again, open-flame burn treatments killed more *C. fluminea* than steam applications. All treatments caused significant mortality in comparison with control groups (all *P* < 0.05).

### Experiment 6: Efficacy of Rake and Steam Treatments for Inducing Mortality of Water-Submerged *C. fluminea*

**Fig. 5:**
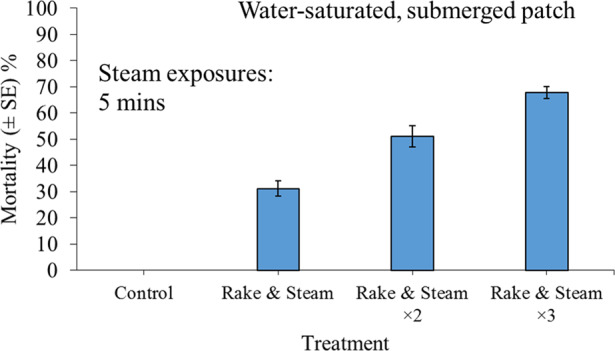
Mean mortality (±SE) of 30 adult *Corbicula fluminea* specimens (480 ind. m^−2^) 24 h post exposure to the application of combined 30-s rake and 2.5-min steam treatments. Specimens resided within 4 cm of completely saturated sand substrate, which was submerged below a 1-cm layer of water. Treatments were performed as single, double (×2) or triple (×3) applications. All treatment combinations were replicated in triplicate, i.e., *n* = 3

There was 100% survival of control specimens, while up to 70% mortality in *C. fluminea* was observed following rake and steam treatments for water-submerged bivalves (Fig. 5). Treatment effects on mortality were significant overall (GLM: *χ*^2^ = 125.88, df = 3, *P* < 0.001), with all rake and steam treatments causing significantly greater mortality than control groups (all *P* < 0.05). Double and triple applications were significantly more effective than single exposures (both *P* < 0.05), whilst differences between double and triple applications were not statistically clear (*P* = 0.11).

### Experiment 7: Direct Steam Exposure for Inducing *D. polymorpha* Mortality

**Fig. 6:**
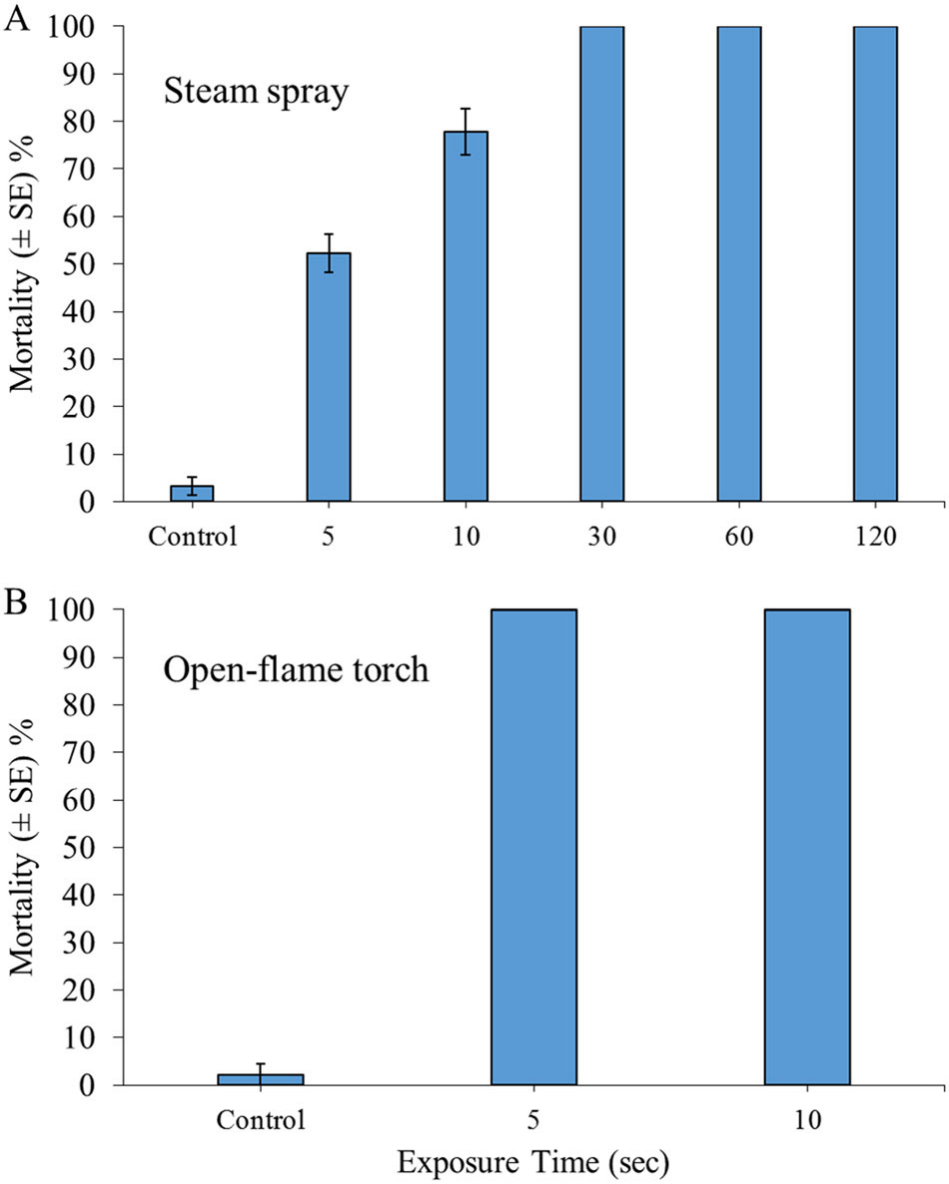
Mean mortality (±SE) of 30 adult *Dreissena polymorpha* at 24 h post exposure to direct steam-spray (≥ 100 °C) (**a**), or open-flame burn treatments (~1000 °C) **b**. All treatments were replicated in triplicate, i.e., *n* = 3

Between 93 and 100% of control specimens were observed to survive, while 100% mortality was consistently recorded in *D. polymorpha* at steam exposures exceeding 30 s (Fig. 6a). Accordingly, the effects of steam exposure were significant on mortality rates (GLM: *χ*^2^ = 388.82, df = 5, *P* < 0.001), wherein all steam-exposure durations were significantly more efficacious than controls (all *p* < 0.001). In turn, 5-s exposures were significantly less effective than longer steam-exposure durations (all *P* < 0.01).

### Experiment 8: Direct Open-flame Exposure for Inducing *D. polymorpha* Mortality

There was between 93 and 100% survival of control specimens, with 100% mortality of *D. polymorpha* being exhibited following flame exposures for both 10- and 30-s applications (Fig. 6b). The effects of open-flame exposures were, therefore, significant overall (GLM: *χ*^2^ = 319.61, df = 2, *P* < 0.001), with both flame-exposure durations driving significantly increased mortality, as compared with control groups (both *P* < 0.001).

## Discussion

Whilst previous studies have highlighted that steam-spray treatments can kill invasive bivalves and decontaminate equipment to prevent their further spread (e.g., Coughlan et al. 2019a, 2020; Joyce et al. 2019), Experiment 1 of the current study has shown that steam-spray treatments, lasting ≥ 60 s, can also rapidly kill *C. fluminea* residing on the surface of sand substrate. It appears that the application of extreme heat, such as steam spray, causes the haemolymph and extrapallial fluid in the treated bivalve to boil, rupturing internal soft-tissue structures (NEC *pers. obs*.). Further, Experiment 2 has highlighted that larger groups of surface-dwelling and buried *C. fluminea* (30 ind. group^−1^, 1179 ind. m^–2^) can also be completely killed following 1-min or 5-min steam exposures, respectively. Although burial of *C. fluminea* in sand substrate can reduce the efficacy of steam treatments, Experiment 3 has shown that disturbing the sediment immediately prior to application of steam can substantially increase *C. fluminea* mortality. In essence, as described by Coughlan et al. (2019b), the repeated raking of substrates containing bivalves churned specimens upwards towards the surface, and likely increased the overall surface area of substrate exposed to steam, whilst also facilitating deeper penetration of the bivalve bed by steam, through the creation of channels and loosening of the substrate. These combined rake and 2.5-min steam treatments caused up to 82% mortality of *C*. *fluminea* following triple applications (30 ind. group^−1^, 480 ind. m^−2^).

Previously, Coughlan et al. (2019b) demonstrated that the application of open-flame heat-torch treatments can be used for effective and substantial population control of *C. fluminea* populations. Here, Experiment 4 has shown longer steam and open-flame burn applications, i.e., thermal shock treatments lasting for a 5-min rather than 2.5-min period, can effectively kill up to 100% of *C. fluminea* specimens (30 ind. group^−1^, 480 ind. m^−2^), following triple applications. Although not statistically evident, open-flame burn treatments tended to kill more *C. fluminea* than steam applications, and this is most likely due to the more intense heat generated by open flame in comparison with steam, i.e., ~1000 °C and ~100 °C, respectively. Therefore, over large areas, the use of open-flame burns could provide for greater population control of *C. fluminea* than steam treatments. Yet, sand substrate also appeared to buffer *C. fluminea* from the more extreme heat of the open-flame torch. Similarly, Experiment 5 has revealed that a deeper substrate depth will reduce the efficacy of rake and thermal shock treatments, for both steam and open-flame applications (30 ind. group^−1^, 480 ind. m^−2^). Further, Experiment 6 has shown that substrate submerged by 1-cm depth of water will also reduce the efficacy of rake and steam treatments (30 ind. group^–1^, 480 ind. m^–2^). Overall, when taken together, these results demonstrate that the application of thermal shock treatments, using steam or open-flame-burn application treatments, can potentially be used for effective, rapid response and substantial population control of *C. fluminea* populations residing within exposed river, lake and canal beds. Similarly, Experiments 7 and 8 have shown that steam-spray and open-flame-burn treatments can also be used to suppress low-water-exposed *D. polymorpha*. In effect, we suggest that thermal shock treatments could form part of a suite of tools available for population control of these, and other, invasive bivalves (e.g., quagga mussel *Dreissena rostriformis bugensis* Andrusov, 1897, golden mussel *Limnoperna fortune* Dunker, 1857). Nevertheless, multiple applications of thermal shock treatments, applied over numerous visits to the invaded site over the course of a number of days or weeks, may be required to achieve meaningful population control, if not complete eradication.

Cold thermal shock treatments, caused by an application of dry-ice pellets, have been shown to effectively kill *C. fluminea* during low-water exposure and at a water depth of up to 10 cm (Coughlan et al. 2018b). However, the application of dry-ice pellets still requires testing under field conditions. Currently, underwater-control methods for bivalve populations, such as dredging and benthic barriers, remain problematic, expensive, labour-intensive and are frequently unsuccessful (e.g., Wittmann et al. 2012a, b; Sheehan et al. 2014). Although the results presented in this study do not offer a direct solution for the in-water management of invasive bivalves, thermal shock treatments could be combined with mechanical dredging methods to improve waste-disposal practices, thus ensuring that any extracted bivalves are completely killed prior to final disposal. For example, to reduce the cost of waste disposal, guaranteed in situ mortality may allow for bivalves to be returned to the waterway following thermal shock treatments, rather than requiring the relocation of biological waste material (Coughlan et al. 2019b). However, although short-term negative non-target ecological impacts can potentially be outweighed by the long-term positive conservation benefits gained by removing damaging invaders (Woodford et al. 2013), a thorough assessment of direct and residual treatment effects on biodiversity, such as mortality of native species and decomposition effects, would be required on a case-by-case basis. In particular, the scale of a bivalve infestation and the extent to which a treatment is applied will govern the gravity of effects on freshwater systems. However, large and highly connected aquatic systems, such as major rivers, will likely better tolerate treatment effects than small isolated waterbodies. Further, due to a current paucity of empirical data, in situ assessments are required to determine the long-term impacts associated with both natural and human-instigated mass die-offs of bivalves on community structure and ecosystem functioning, such as the effect of large pulses of nitrogen and increased oxygen stress, and the accumulation of large numbers of empty bivalve shells (McDowell and Sousa 2019). Nevertheless, given the high levels of biological connectivity and relatively short invertebrate species recolonisation times associated with lotic systems (Yount and Niemi 1990; Caffrey et al. 2010; Wittmann et al. 2012a, b; Coughlan et al. 2017a), rapid recovery may possibly ensue following thermal treatments, and this can be further aided with appropriate management strategies. However, rapid recovery can also occur for invasive species (Wittmann et al. 2012a, b).

Whilst the results presented within this study are promising, additional research is needed to confirm the effectiveness of thermal shock treatments under natural field conditions. In particular, the buffering effects of both deeper and different substrate types, and deeper water submergence, should be assessed. Equally, further assessment will need to be given to the clustering and layering, whereby outer layers may shield underlying mussels at higher densities (Coughlan et al. 2019b). Examination of synergistic approaches, whereby multiple applications of various population-control mechanisms could be combined and strategically applied to increase overall bivalve mortality, should also be considered. For example, both hot and cold thermal shock treatments could be combined, which may potentially increase mortality rates through additional thermal shock across a greater temperature gradient (Coughlan et al. 2019b). In addition, the sudden exposure of *C. fluminea* to a hot thermal shock treatment, following an initial dramatic cooling from a prior application of dry ice, may cause substantially greater mortality due to greater differential temperature change (e.g., –78 °C to 1000 °C). Finally, in all cases, the potential impact of thermal shock treatments on non-target organisms inhabiting bivalve beds should also be considered.

Although the application of thermal shock treatments could be expensive and laborious, given the current lack of effective and environmentally friendly invader eradication and population-control methods, the excellent potential shown by these innovative treatments requires further investigation. In particular, a comparative assessment of financial costs of the proposed treatments, current control practices and the cost of inaction should be undertaken. Whilst thermal shock treatments will incur an expense, this may be relatively more affordable than other management strategies, especially if it provides for rapid-reaction and long-term population control. Overall, we argue that thermal shock treatments could possibly represent a relatively more user- and environmentally friendly method of invasive bivalve control, compared with mechanical dredging, biocides or chemical treatments.
